# Increased sialylation of site specific O-glycoforms of hemopexin in liver disease

**DOI:** 10.1186/s12014-016-9125-x

**Published:** 2016-09-21

**Authors:** Miloslav Sanda, Julius Benicky, Jing Wu, Yiwen Wang, Kepher Makambi, Jaeil Ahn, Coleman I. Smith, Peng Zhao, Lihua Zhang, Radoslav Goldman

**Affiliations:** 1 Department of Oncology, Georgetown University, PS Room GD11, 3800 Reservoir Rd NW, Washington, DC 20057 USA; 2 Department of Oncology, Georgetown University, NRB Room E207, 3970 Reservoir Rd NW, Washington, DC 20057 USA; 3 Department of Biostatistics, Bioinformatics, and Biomathematics, Georgetown University, Building D Suite 180 Room 185, 4000 Reservoir Rd NW, Washington, DC 20057 USA; 4 Department of Biostatistics, Bioinformatics, and Biomathematics, Georgetown University, Basic Science Building D Room 255, 3900 Reservoir Rd NW, Washington, DC 20057 USA; 5 MedStar Georgetown University Transplant Institute, 2-PHC, 3800 Reservoir Rd NW, Washington, DC 20057 USA; 6 Complex Carbohydrate Research Center, University of Georgia, Athens, GA USA

**Keywords:** O-glycosylation, Sialic acid, Fibrosis, Cirrhosis, MRM quantification

## Abstract

**Background:**

Non-invasive monitoring of liver disease remains an important health issue. Liver secreted glycoproteins reflect pathophysiological states of the organ and represent a rational target for serologic monitoring. In this study, we describe sialylated O-glycoforms of liver-secreted hemopexin (HPX) and quantify them as a ratio of disialylated to monosialylated form (S-HPX).

**Methods:**

We measured S-HPX in serum of participants of the HALT-C trial using a LC–MS/MS-MRM assay.

**Results:**

Repeated measurements of S-HPX in the samples of 23 disease-free controls, collected at four different time points, show that the ratio remains stable in the healthy controls but increases with the progression of liver disease. The results of measurement of S-HPX in serum of participants of the HALT-C trial show that it increased significantly (Kruskal–Wallis test, p < 0.01) in liver disease as the stage of fibrosis progressed in liver biopsies. We observed a 1.7-fold increase in fibrosis defined as Ishak score 3–4 (24.9 + 14.2, n = 22) and 4.7-fold increase in cirrhosis defined as Ishak score 5–6 (68.6 + 38.5; n = 24) compared to disease-free controls (14.7 + 6.7, n = 23). S-HPX is correlated with AFP, bilirubin, INR, ALT, and AST while inversely correlated with platelet count and albumin. In an independent verification set of samples, S-HPX separated the Ishak 5–6 (n = 15) from the Ishak 3–4 (n = 15) participants with AuROC 0.84; at the same time, the Ishak 3–4 group was separated from disease-free controls (n = 15) with AuROC 0.82.

**Conclusion:**

S-HPX, a measure of sialylated O-glycoforms of hemopexin, progressively increases in fibrotic and cirrhotic patient of HCV etiology and can be quantified by an LC–MS/MS-MRM assay in unfractionated serum of patients. Quantification of sialylated O-glycoforms of this liver secreted glycoprotein represents a novel measure of the stage of liver disease that could have a role in monitoring the progression of liver pathology.

**Electronic supplementary material:**

The online version of this article (doi:10.1186/s12014-016-9125-x) contains supplementary material, which is available to authorized users.

## Background

Hepatitis C virus (HCV) is a leading cause of chronic liver disease in the US [1, 2]. Approximately 20 % of chronic HCV cases develop liver cirrhosis and 2.5 % develop HCC which appears most commonly in cirrhotic livers [3]. While the recently available HCV treatment regimens are expected to eventually decrease the burden of liver disease [4], the large number of unidentified and untreated HCV infections, uncertain reversibility of liver damage, and increasing impact of NAFLD/NASH suggest that non-invasive monitoring of liver disease remains an important clinical issue [3, 5, 6].

Biopsy is the diagnostic standard for monitoring of liver disease in spite of its invasive nature, associated complications, and inconsistent sampling [7]. It is expected that non-invasive detection of liver fibrosis at a potentially reversible stage, improved predictive models of liver disease progression, and detection of resectable early stage HCC will have major impact on disease outcomes [8, 9]. This has stimulated considerable interest in non-invasive serologic, liver function, and imaging tests that could complement biopsy in liver disease classification [10, 11]. In spite of these efforts, alternatives to biopsy have not yet reached general acceptance and the search for viable options continues. We have therefore designed new serologic assays for monitoring of unusual glycoforms of liver secreted proteins [12, 13] and evaluated, for the first time, the potential of sialylated O-glycoforms of hemopexin (HPX) in serologic monitoring of liver disease.

Glycosylation is a common and diverse modification of proteins secreted by the liver. Two major pathways of glycosylation of secretory proteins have been described: (1) N-glycosylation of asparagine (Asn) in the sequence Asn-X-Ser/Thr (X ≠ Pro); and (2) mucin-type O-glycosylation of serine or threonine (Ser/Thr) without a well-defined sequence motif. Diversity and functions of the proteome are substantially expanded by these modifications [14, 15]. We and others have shown that changes in protein glycosylation accompany the development of fibrosis, cirrhosis, and HCC [16–19]. Unusual N-glycoforms of immunoglobulins as well as liver secreted proteins have been proposed as serologic tests for the monitoring of liver disease [16, 20, 21]. Methodological improvements show that O-glycosylation of proteins is as common and important as N-glycosylation [22]; however, O-glycosylation of liver secreted proteins remains largely unused in serologic assays of liver disease. We have therefore examined a common type of O-glycosylation, the GalNAc α Ser/Thr linkage, known as mucin type O-glycosylation [23]. We do this because mucin type O-glycans have profound functional impact [24, 25] and are associated with a wide range of proteins, including liver secreted glycoproteins which can be followed by serologic assays [26–28]. The truncated O-glycans of Tn and STn antigens, one of the well-known examples of disease-associated O-glycoforms [29], affect gastrointestinal (patho)physiology and are increased in tumors [30, 31]. However, the structural and functional variants of mucin type O-glycoforms associated with specific proteins and disease processes only begin to be understood [5, 32]. We have therefore selected HPX as a model liver secreted glycoprotein to study changes in the detectable site-specific O-glycoforms in liver disease.

HPX is secreted mainly by hepatocytes, binds heme in the blood stream, and the complex is cleared from circulation by a hepatocyte-specific membrane receptor [33]. It was reported that serum concentration of HPX [0.4–1.5 g/L] does not vary significantly in liver disease [15]. While N-glycosylation of HPX is altered in liver disease [21, 34, 35], O-glycosylation of HPX was not to our knowledge examined in this context. Wada et al. [26] documented that at least three O-glycosylation sites at the N-terminus of HPX carry O-glycans and that the N-terminal threonine of human HPX is consistently and fully occupied by the mucin type O-glycans. Because of low variability of this O-glycosylation of HPX, the authors proposed that it could serve as a new marker of congenital disorders of glycosylation [26]. We therefore examine O-glycoforms of HPX at progressing stages of liver disease of HCV etiology. To this end, we have developed targeted LC–MS/MS-MRM methods for quantification of the site-specific O-glycoforms of HPX and applied them to the study of HPX samples of participants in the hepatitis C antiviral long-term treatment against cirrhosis trial (HALT-C).

## Patients, materials and methods

### Study population

The initial discovery of O-glycoforms of HPX associated with liver disease progression was carried out using samples of HCC patients (n = 10), cirrhotic patients (n = 10), and healthy individuals (n = 10) enrolled in collaboration with the Department of Hepatology and Liver Transplantation, Georgetown University Hospital, Washington, DC under protocols approved by the Institutional Review Board as described previously [36]. Briefly, all patients (HCC and cirrhosis) selected for this study had chronic HCV infection as primary diagnosis and all the HCC participants had stage 1 and 2 tumors according to the 7th edition of the American Joint Committee on Cancer Staging manual. Blood samples were collected using EDTA Vacutainer tubes (BD Diagnostics, Franklin Lakes, NJ, USA); plasma was collected according to the manufacturer’s protocol and was stored at −80 °C until use. Participants were split into groups of five and plasma was pooled for analysis (two pools per group, five participants per pool). All the groups were matched on age, race (60 % Caucasian, 40 % African-American), and gender (80 % males); HCC and cirrhosis groups were further matched on MELD score as described in the previous literature [36].

Further serum samples were provided by NIDDK from the HALT-C trial, a prospective randomized controlled trial of 1050 patients that evaluated effect of long-term low-dose peginterferon alpha-2a in participants that failed initial anti-HCV therapy with interferon [37, 38]. A total of 114 participants (76 liver disease patients from the HALT-C trial and 38 disease-free controls recruited at Georgetown University) (35) were included in the analysis (Table 1). Liver disease status of the HALT-C participants was classified based on biopsy-evaluation into fibrosis (Ishak score 3–4, n = 37) or cirrhosis (Ishak score 5–6, n = 39) groups. To minimize classification error, we selected patients with consistent Ishak scores at three biopsy measurements at month 0, 24, and 48 after randomization. However, two fibrotic participants with Ishak 3 had only one biopsy and seven participants (three cirrhotic and four fibrotic) crossed disease categories at one biopsy time point. For those participants, we analyzed blood samples collected at a time point closest to the disease outcome consistent with their group assignment. The two groups of liver disease participants and the controls were matched on age and gender. The disease-free participants donated blood samples at four successive visits in the span of 1 year and answered a brief questionnaire inquiring about basic demographic information and medical history. The study groups were further divided into discovery (23 disease-free controls, 22 fibrosis, and 24 cirrhotic) and verification (15 disease-free controls, 15 fibrotic, and 15 cirrhotic) sets of participants (Additional file 1). The fibrosis and cirrhosis groups in the discovery sets consist of similar proportions of participants from the IFN treatment and control arms of the HALT-C trial; the verification set consist of participants from the control arm.

**Table 1:** Basic characteristics of disease-free controls and groups of the HALT-C participant

	Healthy	Fibrosis	Cirrhosis	*p* value^a^
N	38	37	39	n/a
Male (%)	68	59	72	0.502
Race (CA/AA)	20/12^b^	30/7	28/11	0.106
Age	49.3 ± 7.7	50.1 ± 7.1	50.9 ± 6.5	0.614
IFN treatment (yes/no)	n/a	13/24	12/27	0.686
ALT (U/L)	n/a	89.1 ± 41.3	129.7 ± 69.5	0.007
AST (U/L)	n/a	67.4 ± 25.6	110.0 ± 65.0	0.002
Platelet (10^9^/L)	n/a	191.2 ± 59.4	144.7 ± 57.4	<0.001
Albumin (g/dL)	n/a	4.0 ± 0.3	3.8 ± 0.4	0.115
AFP (ng/mL)	n/a	11.7 ± 14.7	22.9 ± 24.6	0.005
Alkaline phosphatase (U/L)	n/a	91.7 ± 29.6	123.6 ± 52.1	0.636
INR	n/a	1.0 ± 0.1	1.1 ± 0.1	<0.001
Bilirubin (mg/dL)	n/a	0.7 ± 0.4	0.9 ± 0.4	0.039
Creatinine (mg/dL)	n/a	0.8 ± 0.1	0.9 ± 0.2	0.936
MELD	n/a	6.8 ± 1.3	7.5 ± 1.4	0.006
APRI	n/a	0.9 ± 0.5	2.4 ± 2.5	<0.001
FIB-4	n/a	2.1 ± 1.1	3.5 ± 2.7	<0.001

Values are expressed as mean ± SD

^a^ *p* value was calculated using Mann–Whitney U test or Kruskal–Wallis test for quantitative variables and Chi square test for categorical variables

^b^Six healthy controls belong to the Hispanic ethnic group

### Isolation of HPX and separation of glycopeptides by HILIC chromatography

HPX was purified from plasma by hemin affinity followed by C18 chromatography as described previously [12]. Isolated HPX (20 pmol), dried in a vacuum concentrator and stored at −80 °C, was suspended in 50 mM ammonium bicarbonate solution, pH 7.8 (Sigma-Aldrich, St. Louis, MO, USA) with 0.05 % RapiGest (Waters, Milford, MA, USA), reduced, alkylated, and digested with 0.2 µg of Trypsin Gold for MS (Promega, Madison, WI, usa) in a Barocycler reactor (Pressure BioSciences, South Easton, MA, USA) at 37 °C for 60 min as described [12]. O-glycopeptides were isolated by HILIC chromatography on a ZIC-HILIC column 150 × 2.1 mm, 3 μm particles (EMD Merck, Germany) in a 30 min HPLC (Agilent, Santa Clara, CA, USA) gradient of ACN/H_2_O with 0.01 % TFA. Starting conditions were set to 90 % ACN with 0.01 % TFA followed by 30 min gradient elution to 90 % of H_2_O with 0.01 % TFA. Peptides were monitored at 214 and 280 nm using a DAD detector (Agilent Technologies, Santa Clara, CA, USA). Glycopeptide fractions were collected and dried on a vacuum concentrator (Labconco, Kansas City, MO, USA) for further use.

### De-sialylation and exoglycosidase treatment of the O-glycopeptides of HPX

Tryptic digest of HPX or the HILIC-enriched glycopeptide fractions of the tryptic digest were de-sialylated with 2 M acetic acid (Sigma-Aldrich) at 80 °C for 2 h. Completion of the de-sialylation reaction was confirmed by LC–MS/MS analysis (Additional file 1). De-sialylated glycopeptides were evaporated in a vacuum concentrator (Labconco) and further treated with 0.1 U of 1–3 beta galactosidase (New England BioLabs, Ipswich, MA, USA) in 50 mM sodium acetate, pH 6, at 37 °C for 8 h. Products of the reaction were cleaned for further analysis by SPE C18, concentrated using vacuum concentrator, and dissolved in 0.1 % formic acid for further LC–MS analysis.

### LC–MS/MS analysis

Tryptic digest of HPX, HILIC-enriched fractions, or acid- and exoglycosidase-treated glycopeptide fractions were analyzed under identical chromatographic conditions. Peptides were separated by reversed phase chromatography (Tempo Eksigent-AB Sciex, Framingham, MA, USA) on a ChromXP C18-CL (3 μm, 120Å, 180 μm, 20 mm) trap column and ChromXP C18-CL (3 μm, 120 Å, 75 μm, 150 mm) HPLC capillary chip column (Eksigent-AB Sciex) interfaced with a 5600 TripleTOF mass spectrometer (AB Sciex, Framingham, MA, USA). Chromatographic method consisted of 10-min trapping/washing step [2 % ACN, 0.1 % formic acid (FA)] at 3 μL/min flow rate and 30 min gradient elution at a flow rate of 300 nL/min (solvent A: 2 % ACN with 0.1 % FA; solvent B: 100 % ACN with 0.1 % FA) using the following timetable: 5–45 % solvent B 0–23 min; 45–100 % solvent B 23–25 min; 100 % solvent B 25–30 min. Mass spectrometer was set to ion spray voltage 2400 V, ion source gas (GS1) 13, declustering potential 90 and interface heater temperature 150 °C, and operated in data-dependent mode; each survey scan (*m*/*z* 400–1600) was followed by MS/MS on 25 precursor ions in the range *m*/*z* 100–1800, with the dynamic exclusion time set to 6 s, and 150 counts threshold for two repeated precursors. Collision energy was set automatically according to charge state and m/z of precursor ion. Data depended analysis was used to identify major glycoforms and precursor XIC of 0.05 Da window was used to evaluate changes in glycoform quantities in liver disease (cirrhosis and HCC). Glycopeptide intensities were normalized to an internal tryptic peptide of HPX to eliminate influence of changes in the concentration of HPX protein on the quantitative outcome.

### LC/MS3 and LC–MS/MS-MRM analysis of the O-glycopeptides of HPX

Studies of S-HPX in serum of patients were done directly without enrichment of HPX. Serum samples (2 µL) were diluted in 140 µL of 25 mM NH_4_HCO_3_ with 0.1 % *Rapi*Gest (Waters), reduced by 5 mM DTT for 1 h at 60 °C, and alkylated with 15 mM IAA for 20 min at room temperature in the dark. Residual IAA was reduced with 5 mM DTT for 20 min at RT. Reduced and alkylated samples (20 μL) were digested with trypsin in a Barocycler reactor as described above. Tryptic peptides were analyzed on a NanoAcquity UPLC (Waters) by RP chromatography on a symmetry C18 (3 µm, 180 µm, 20 mm) trap column and UPLC capillary column (BEH 300Å, 1.7 µm, 150 mm × 0.75 µm) (Waters) interfaced with a 6500 QTRAP mass analyzer (AB Sciex). Separation was achieved by a 60 min gradient elution with ACN containing 0.1 % formic acid (B) starting from 2 % ACN with 0.1 % formic acid (A) (0–1 min 99 % A, 1–20 min 99–50 % A, 20–38 min 50–2 % A, 38–45 min 2 % A, 45–45.5 min 2–99 % A, 45.5–60 min 99 % (A). Ion source parameters were set to declustering potential 80 V, curtain gas 20, ion spray voltage 2400 V, ion source gas1 22, interface heater 180 °C, entrance potential 10 V, collision exit potential 13 V. We compared directly the MS/MS-MRM monitoring to MS3 monitoring. The MS/MS-MRM measured the ratio of two analytes, the monoisalo-T glycopeptide to Y0 fragment transition (843.9 → 905.6) and the disialo-T glycopeptide to Y0 transition (916.7 → 905.6). The LOQ (S/N >10) of the monoisalo-T glycopeptide to Y0 fragment transition is 5 fmol on column based on the addition of a stable isotope labeled glycopeptide standard. We could not determine absolute value of LOQ for the disialo-T glycopeptide because the synthetic standard is not available. However, the S/N of the transition was >10 for all the samples examined. All parallel measurements of S-HPX were below 20 % RSD. The MS3 assay monitored THREE product ions (m/z: 504.1, 716.3 and 802.5) in the ion trap MS3 spectra with window width 3 Da; these three peptide backbone fragments derive from the peptide ion obtained in MS2 of the respective glycopeptide precursor ions as described (12). IT scan time of 2000 Da/s, Q0 trapping and 20 ms fixed LIT fill time were used for the MS3 method. A QC sample (one control serum sample) was prepared as described above and injected once per two sample injections in order to evaluate method stability and reproducibility. Data analysis was carried out using MultiQuant software 2.1 (AB Sciex).

### Statistical analysis

The ratio of disialylated (NeuAc_2_Hex_1_HexNAc_1_) to monosialylated (NeuAc_1_Hex_1_HexNAc_1_) glycoforms of the peptide TPLPPTSAHGNVAEGETKPDPVTER of HPX carrying one O-glycan on the T1 (S-HPX) was used as a final quantitative measure for evaluation of liver disease. S-HPX in the disease-free controls (n = 38) was compared to the fibrosis (n = 37) and cirrhosis (n = 39) groups of participants in the HALT-C trial divided into and verification sample sets. All samples were blinded to the analyst and randomized in order of analysis. Descriptive statistics of participants within the three disease groups are summarized in Table 1; Chi square tests were used for categorical variables and Mann–Whitney U test or Kruskal–Wallis test were for quantitative variables. S-HPX between the three disease groups in the discovery set (Fig. 3a), S-HPX between four different blood draws in the disease-free controls (Fig. 3b), and influence of IFN treatment on S-HPX (Additional file 1) were analyzed using Mann–Whitney U test or Kruskal–Wallis due to non-normality of S-HPX values. Logistic regression models were used to evaluate the association between S-HPX and liver disease status in the discovery set. Disease status was the dependent variable and S-HPX was the main independent variable; other clinical variables including AFP and platelet count were used as independent variables. Model estimates can be found in Additional file 1. Optimal cut-off point for each independent variable was determined by maximizing sensitivity and specificity. The performance of S-HPX and other variables in classification of liver disease was assessed using area under the receiver operating characteristic curve (AuROC) analysis in the verification set (Table 4) using the cut-off points derived from the discovery set. All reported *p* values are two sided. Statistical analyses were performed using SAS v 9.4 (SAS Institute, Cary, NC, USA).

## Results

### O-glycopeptides of HPX in liver disease

**Fig. 1:**
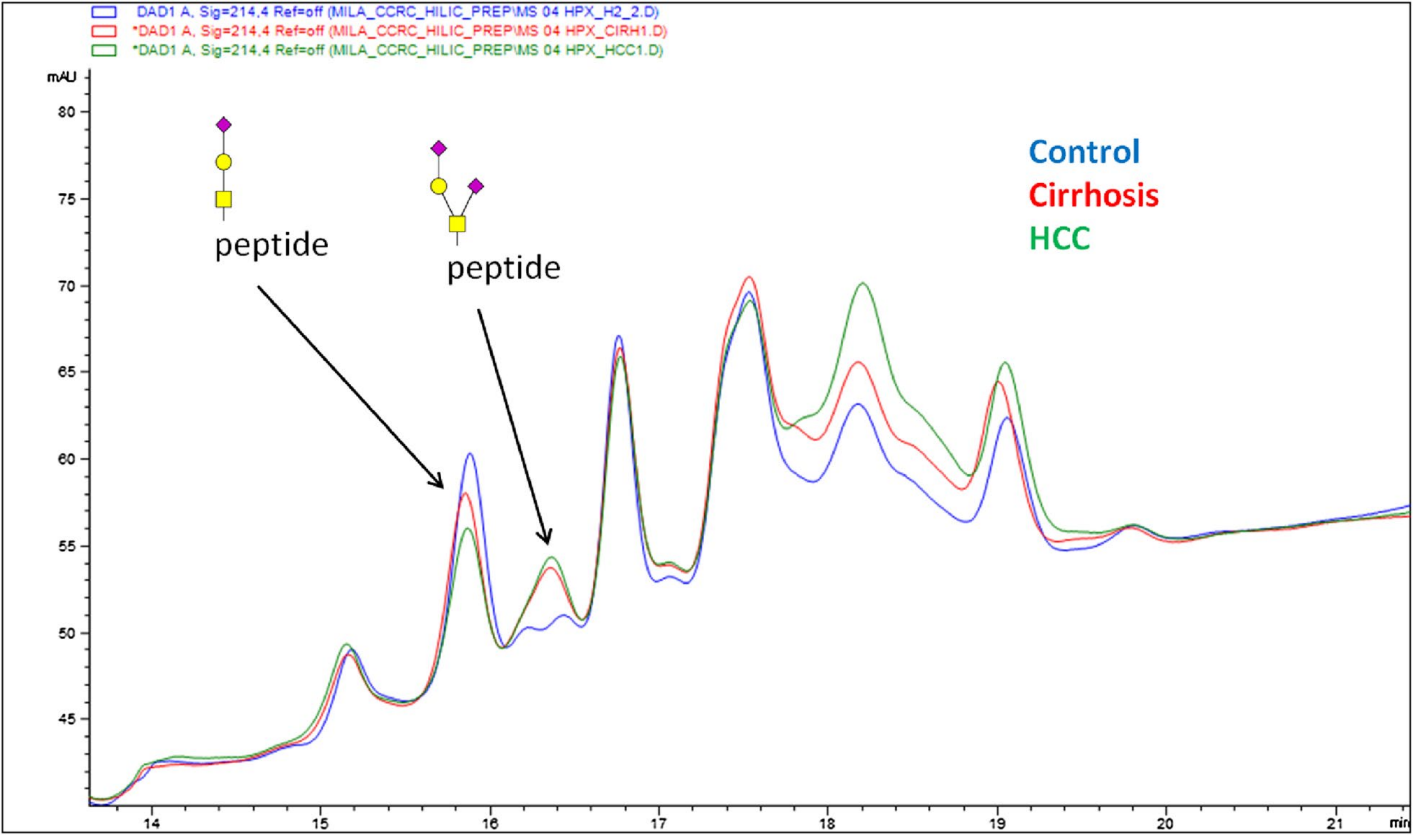
Peaks of the O-glycopeptides of HPX separated by HILIC chromatography. Peaks correspond to a tryptic digest of hemopexin (HPX) isolated from the plasma of controls (*blue*), cirrhotic (*red*) and HCC (*green*) patients. The peaks indicated by *arrows* represent the mono-sialylated core 1 O-glycopeptide (RT 15.9 min, m/z 843.6) followed by the di-sialylated core 1 O-glycopeptide (RT 16.4 min, m/z 916.4)

**Fig. 2:**
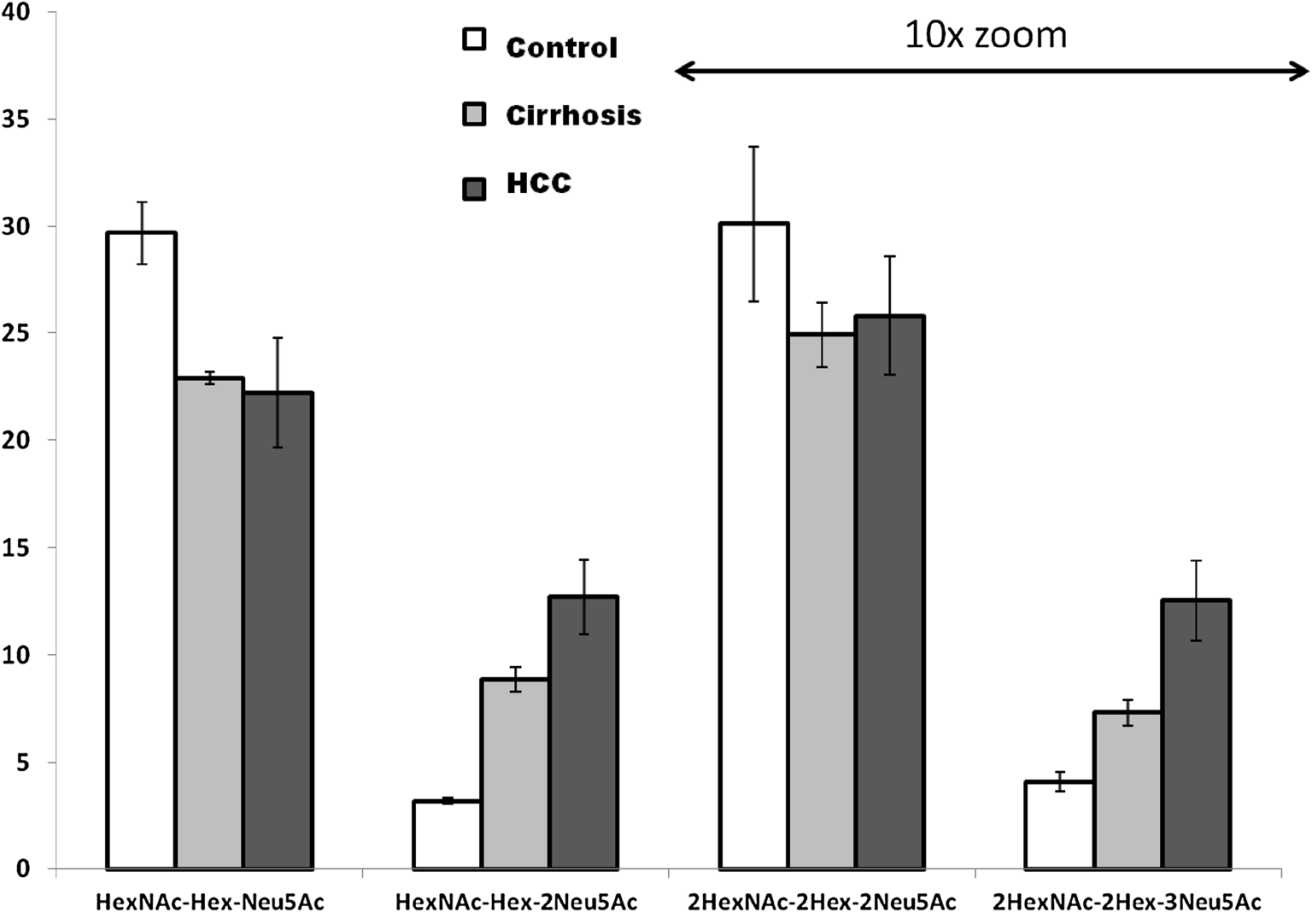
Quantification of the mono- and bis- sialylated O-glycopeptides of HPX. Quantification of mono- (HexNAc-Hex-Neu5Ac and 2HexNAc-2Hex-2Neu5Ac) and bis- (HexNAc-Hex-2Neu5Ac and 2HexNAc-2Hex-3Neu5Ac) sialylated O-glycopeptides of HPX isolated from pooled plasma of healthy volunteers (*white bars*), cirrhotic (*grey bars*), and HCC (*black bars*) patients. Intensities of the peptides, doubly O-glycosylated at T1 and T6 (right side of the figure), are ×10 magnified. *Results* represent XIC of precursor ions normalized to an internal peptide of HPX and *error bars* represent analysis of two different plasma pools (samples of five participants in each pool) as described in “Patients, materials and methods” section

Plasma samples from disease-free controls and cirrhotic patients (CIR) with or without HCC provided a baseline for our examination of O-glycoforms of HPX detectable in liver disease. HPX was isolated by hemin affinity and C18 HPLC from the pooled plasma of participant as described in “Patients, materials and methods”. The yield of HPX, purified to >95 % purity, was 20–25 µg per 100 µL of plasma. Glycopeptides of HPX were enriched by HILIC chromatography (Fig. 1) and analyzed by LC–MS/MS as described in [12, 19]. Combined analysis of all the pools led to the identification of 15 O-glycopeptides of HPX (Table 2) derived from the N-terminal tryptic peptide of HPX, TPLPPTSAHGNVAEGETKPDPVTER. We did not observe other O-glycosylated peptides of HPX in any of the samples. Estimates of the abundance of these O-glycoforms, based on intensities of precursor ions in the LC–MS/MS scans, showed that two glycoforms (HexNAc-Gal-Neu5Ac, 66 % total intensity and HexNAc-Gal-2Neu5Ac, 20 % total intensity) dominated the distribution. These two glycoforms were also clearly visible in the HILIC chromatograms by UV–Vis detection (Fig. 1). The chromatogram showed that the doubly sialylated glycoform (HexNAc-Gal-2Neu5Ac) increases in cirrhosis and HCC samples (red and green trace) compared to disease-free controls (blue trace) while the mono-sialylated O-glycoform (HexNAc-Gal-Neu5Ac) decreases. Major contribution of the above glycoforms to these isolated peaks was confirmed by LC–MS/MS analysis. Further analysis of the precursor ion peak-intensities in XIC of the LC–MS data confirmed the trend towards increased sialylation of the O-glycoforms of HPX in liver disease (Fig. 2). The results show an approximately fourfold increase in disialylated O-glycopeptide of HPX in cirrhosis and further increase in the cirrhotics with HCC with a simultaneous approximately 20 % decrease in the monosialylated O-glycopeptide of HPX. The changes are quite consistent in measurements of two independent pools of samples per group. Figure 2 also shows that the minor O-glycoforms (2HexNAc-2Gal-2Neu5Ac and 2HexNAc-2Gal-3Neu5Ac), which are about ten-times less intense, show a similar trend towards increased sialylation in the liver disease context.

**Table 2:** Detected glycan compositions of the TPLPPTSAHGNVAEGETKPDPVTER O-glycopeptides in tryptic digest of hemopexin isolated from pooled plasma of disease-free controls, cirrhotic, and HCC patients

m/z	Delta (ppm)	Glycan composition	XIC (%)
973.5 (3+)	9.55	HexNAc	0.35
770.9 (4+)	2.08	HexNAc-Gal	1.74
843.6 (4+)	3.20	HexNAc-Gal-Neu5Ac	66.19
916.4 (4+)	3.38	HexNAc-Gal-2Neu5Ac	19.61
894.4 (4+)	−0.67	2HexNAc-Gal-Neu5Ac	0.16
934.9 (4+)	3.85	2HexNAc-2Gal-Neu5Ac	0.38
1007.7 (4+)	3.27	2HexNAc-2Gal-2Neu5Ac	7.22
1080.5 (4+)	4.54	2HexNAc-2Gal-3Neu5Ac	1.62
1153.2 (4+)	5.81	2HexNAc-2Gal-4Neu5Ac	0.68
1058.5 (4+)	−5.29	3HexNAc-2Gal-2Neu5Ac	0.09
1099.0 (4+)	6.10	3HexNAc-3Gal-2Neu5Ac	0.15
937.6 (5+)	3.73	3HexNAc-3Gal-3Neu5Ac	1.23
995.8 (5+)	0.40	3HexNAc-3Gal-4Neu5Ac	0.45
1054.0 (5+)	1.99	3HexNAc-3Gal-5Neu5Ac	0.08
1112.3 (5+)	11.60	3HexNAc-3Gal-6Neu5Ac	0.04

Relative abundance is estimated as percent XIC signal of each glycopeptide (m/z) normalized to the sum of all O-glycopeptides

We carried out LC-ETD/MS analysis of the enriched glycopeptides to understand whether the minor glycoforms represent large O-glycans attached to a single site or smaller O-glycans attached to several serines or threonines in the peptide sequence. This technique allows fragmentation of the peptide backbone with preservation of the peptide-glycan bond which allows determination of sites occupied by the O-glycans [28]. To further increase efficiency of our analysis, we labeled the glycopeptides with mTRAQ reagent which increased charge state of the labeled peptides to (4+) and improved ETD fragmentation compared to the unlabeled glycopeptide in charge state (3+). Our analysis was further optimized by removal of sialic acids from the microheterogeneous peptide population by treatment with acetic acid [26] which improved recovery of the informative fragments (Additional file 1). The analysis confirms that we observe smaller O-glycans on multiple serines/threonines. We observe glycosylation on serine or threonine other than T1 only when T1 is occupied which shows that O-glycosylation of this peptide starts from the N-terminus and progresses to additional sites in the C-terminal direction. The additional sites are modified to a lesser degree than the T1 site. Fragmentation of the peptide shown in Additional file 1 documents that position T1 and T6 are occupied in case of doubly glycosylated peptide; triply glycosylated peptide is occupied at position T1, T6, and S7. This was further suggested by the lack of larger glycans in the analysis of permethylated O-glycans detached by beta elimination (data not shown).

### Analysis of S-HPX in serum samples of the HALT-C trial participants

Mono- and di- sialylated core-1 mucin type O-glycans attached to the T1 position of the N-terminal tryptic peptide of HPX were the dominant liver secreted glycoforms (Table 2). These two glycoforms change substantially in liver disease (Fig. 1) with several-fold increase in the disialylated O-glycoform of HPX and simultaneous approximately 20 % decrease in the monosialylated O-glycoform of HPX (Fig. 2). We have therefore selected ratio of the two major glycoforms, which we call S-HPX, as a quantitative measure for assessment of liver disease by our targeted LC/MS3 or LC–MS/MS-MRM assays [12]. Quantification of S-HPX was applied to serum samples of participants in the HALT-C trial (n = 76) and to disease-free controls (n = 38). As expected, the clinical variables including ALT, AST, bilirubin, INR, albumin, AFP, and platelet count significantly differ between the fibrosis and cirrhosis groups (Table 1). Likewise, the calculated MELD score, APRI and FIB-4 indexes significantly differ between these two groups. The participants were further divided into discovery (23 disease-free controls, 22 fibrosis, 24 cirrhosis) and verification (n = 15 each of the three groups) sets of samples (Additional file 1).

**Fig. 3:**
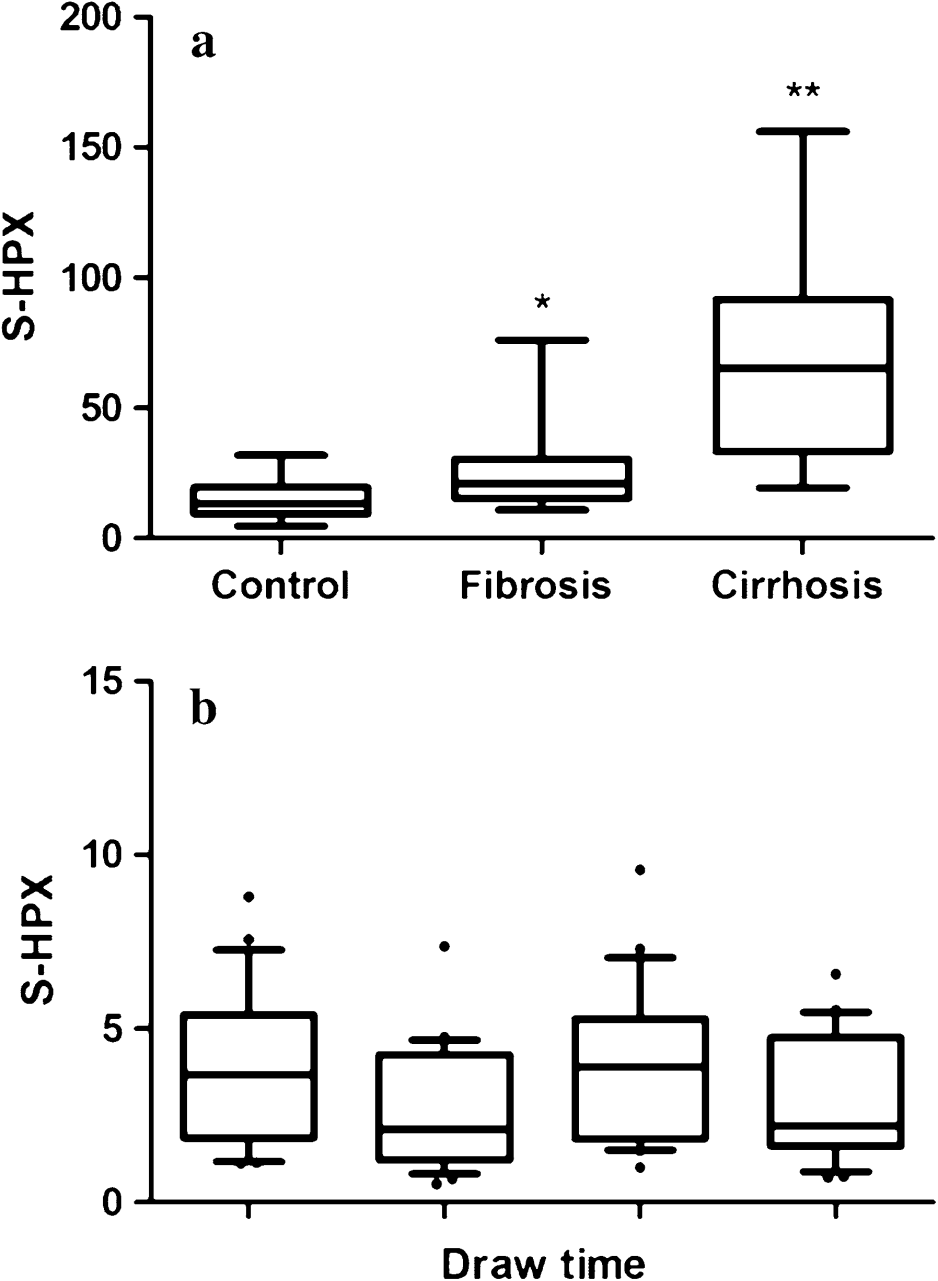
Direct quantification of S-HPX at progressing stages of liver disease. Direct quantification of S-HPX in samples of the following groups of controls and HALT-C participants in the discovery sample set: **a** disease-free controls (n = 23), liver fibrosis (n = 22), and cirrhosis (n = 24) patients; **b** 23 healthy volunteers at four different blood draw times. The *box* and *whisker plots* represents median, 25th–75th percentile, range of values, and outliers; *significantly different at p < 0.01 from control and **from the fibrosis group

Quantification of S-HPX in the discovery set shows a significant increase in the fibrotic HALT-C participants compared to disease-free controls and further increase in the cirrhotic patients (Mann–Whitney U test, *p* < 0.001) (Fig. 3a). At the same time, analysis of S-HPX in disease-free controls (n = 23) that provided samples at four different times in the span of a year showed consistently low S-HPX without significant differences between the draw times (Fig. 3b). Summary statistics of the S-HPX quantification in both the discovery and the verification sets of samples are provided in Additional file 1. We did not observe significant influence of age, gender or race on S-HPX in any of the groups (Additional file 1). To evaluate influence of IFN treatment on S-HPX, we selected a similar proportion of participants in the IFN treatment and control arms of the HALT-C trial in the discovery set. Our analysis shows a trend to lower S-HPX in the IFN treatment arm in both the fibrotic and cirrhotic groups but the changes do not reach significance (Additional file 1). Since the low dose interferon treatment is not clinically used we included only patients from the control arm of the HALT-C trial in the verification set.

Correlation analyses of clinical and demographic variables with S-HPX in the discovery set show multiple significant associations of weak to moderate strength (Table 3). S-HPX is correlated with AFP (r_s_ = 0.58; p < 0.001), bilirubin (r_s_ = 0.41; p = 0.008), INR (r_s_ = 0.39; p = 0.012), ALT (r_s_ = 0.34; p = 0.031), and AST (r_s_ = 0.52; p = 0.001) while inversely correlated with platelet count (r_s_ = −0.56; p < 0.001) and albumin (r_s_ = −0.44; p = 0.004). This means that S-HPX is also positively correlated with the FIB-4 (r_s_ = 0.61; p < 0.001) and APRI (r_s_ = 0.56; p < 0.001) indexes based on combinations of the above variables. These associations are significant to a greater degree in the cirrhotic patients (Table 3). Scatter plots of the variables significantly associated with S-HPX document further these relationships (Additional file 1).

**Table 3:** Associations between clinical variables and S-HPX

	All (n = 46)		Fibrosis (n = 22)		Cirrhosis (n = 24)	
	r_s_	*p* value	r_s_	*p* value	r_s_	*p* value
Age	−0.034	0.832	0.160	0.489	0.017	0.945
Bilirubin (mg/dL)	0.409	0.008*	0.088	0.705	0.393	0.086
Creatinine (mg/dL)	−0.166	0.270	0.129	0.568	−0.363	0.081
INR	0.390	0.012*	0.039	0.866	0.006	0.979
MELD	0.279	0.061	0.215	0.338	0.134	0.534
AFP (ng/mL)	0.578	<0.001*	0.429	0.052	0.439	0.053
Albumin (g/dL)	−0.436	0.004*	−0.040	0.862	−0.511	0.021*
Alk. phosphastase (U/L)	0.195	0.193	0.116	0.608	0.185	0.387
ALT (U/L)	0.337	0.031*	−0.163	0.480	0.346	0.135
AST (U/L)	0.520	0.001*	−0.087	0.709	0.747	0.001*
Platelet (10^9^/L)	−0.564	<0.001*	−0.462	0.035*	−0.358	0.121
FIB-4	0.612	<0.001*	0.366	0.103	0.574	0.008*
APRI	0.562	<0.001*	0.158	0.493	0.589	0.006*

Spearman correlation coefficients were evaluated in all samples combined and in the fibrosis and cirrhosis groups, separately

* Significance level <0.05

**Fig. 4:**
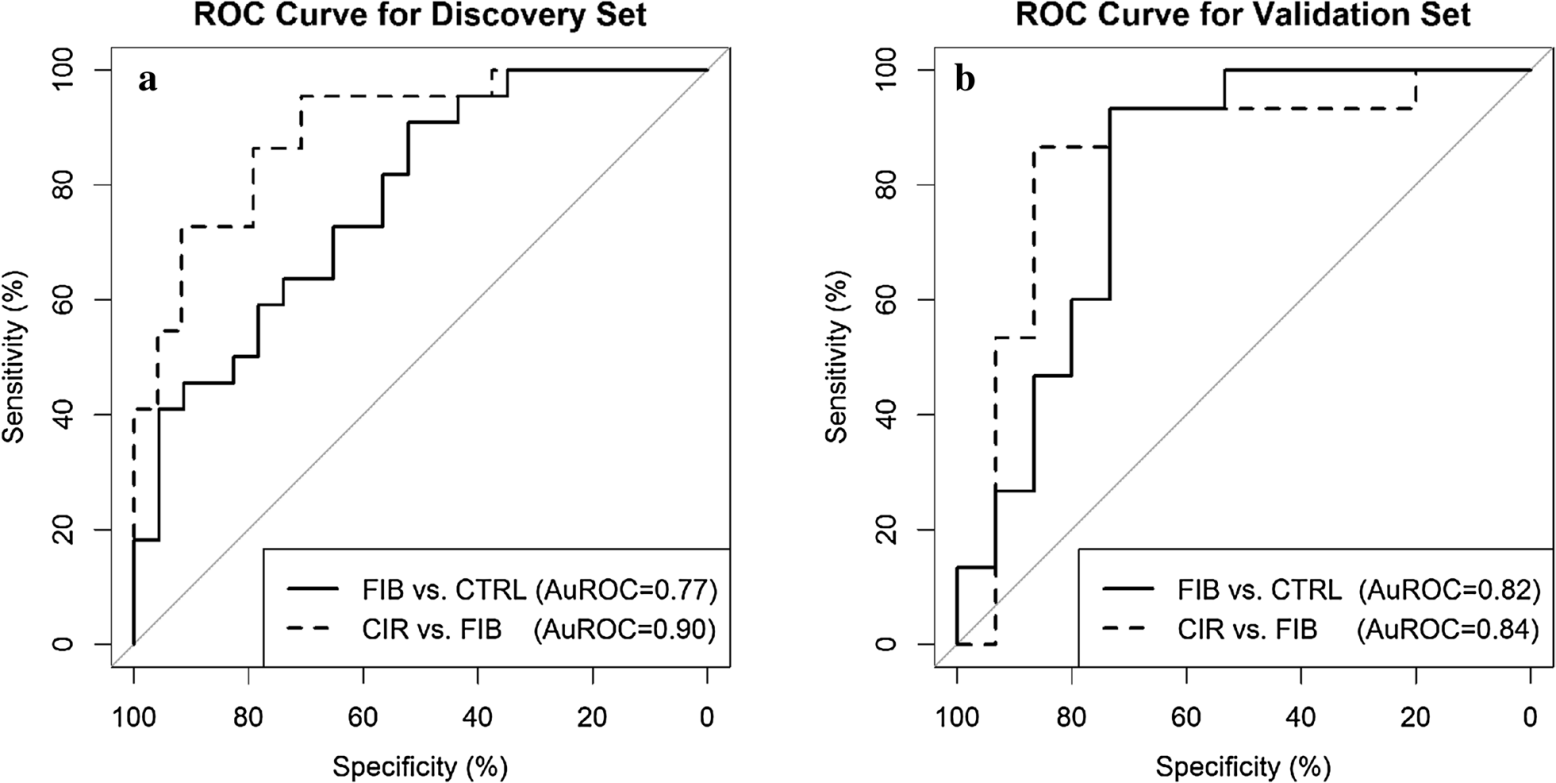
ROC curve analysis of progressing liver disease. ROC curves comparing the following groups based on measurement of S-HPX: **a** discovery: control (n = 23) versus fibrosis (n = 22) and fibrosis (n = 22) versus cirrhosis (n = 24); **b** validation: control (n = 15) versus fibrosis (n = 15) and fibrosis (n = 15) versus cirrhosis (n = 15)

A simple logistic model using S-HPX as an independent variable was used to determine the cutoff values of S-HPX for separation of the healthy, fibrotic, and cirrhotic participants in the discovery set. The resulting model performs well with AuROCs of 0.77 and 0.90 in separating patients who developed fibrosis from the controls and cirrhotic from the fibrotic patients, respectively (Fig. 4a). When the S-HPX cut-off between the fibrotic and control groups was set at 13.5, sensitivity is 91 % and specificity is 52 %. With the cut-off point 42.5 between cirrhotic and fibrotic patient, sensitivity is 96 % and specificity is 71 %.

The diagnostic performance of S-HPX was further tested using 45 verification samples using the LC–MS/MS-MRM measurement of S-HPX and cutoffs determined in the discovery set. The results show an AuROC 0.82 (Fig. 4b) for the separation of fibrotic and disease-free groups, which is better than the AuROC of 0.77 in the discovery set. Here we cannot compare S-HPX to other measures of fibrosis due to lack of other variables in controls. However, diagnostic performance of platelet count, AFP, and the FIB-4 and APRI indexes were compared with S-HPX in distinguishing the cirrhotic patients from the fibrotic patients. S-HPX achieves AuROC = 0.84 which is higher than any other measures with sensitivity and specificity of S-HPX being 60 and 93 %, respectively (Table 4).

**Table 4:** ROC models comparing influence of clinical variables determined at baseline visit on separation of the fibrosis and cirrhosis groups in the validation set

	Cut-off point^a^	Sensitivity (%)	Specificity (%)	AuROC	SE	95 % CI
AFP	11.6	50	79	0.65	0.153	(0.35, 0.95)
Platelet	163	80	50	0.63	0.134	(0.37, 0.89)
APRI	1.45	60	86	0.73	0.145	(0.44, 1.00)
FIB-4	2.60	80	71	0.74	0.144	(0.47, 1.00)
S-HPX	42.5	60	93	0.84	0.145	(0.53, 1.00)

Samples were used in Fig. 4b

^a^Optimal cut-off point was calculated based on fitted models from the discovery set

## Discussion

Non-invasive methods including liver imaging, elastography, and serologic monitoring increasingly complement liver biopsy in diagnosis of fibrotic changes associated with chronic liver diseases [5–9]. Ultrasound elastography is perhaps the fastest developing non-invasive method [7] but glycosylation of liver secreted proteins is an attractive strategy for serologic monitoring of liver disease [13, 16, 17, 19–21]. N-glycosylation of proteins has been explored in detection of pre-malignant liver disease [16, 20] or HCC [17, 21, 39]. AFP-L3, the core-fucosylated N-glycoform of AFP, is an established marker of HCC risk [39] and Debruyne et al. [21] showed that alpha-1,3-fucosylated triantennary N-glycans associated with hemopexin increase in HCC compared to the disease-free, fibrotic, and cirrhotic patients. GlycoFibroTest measures increase in core fucosylated bisecting N-glycans, detached enzymatically from proteins, to detect progressive stages of liver fibrosis [16]. A number of liver secreted glycoproteins have been shown to carry abnormal N-glycans, especially fucosylated glycans [17, 36]. However, O-glycosylation of proteins has not been to our knowledge explored in connection with liver disease.

Our study examines, for the first time, site-specific O-glycoforms of HPX in liver disease (Table 2). We detect 15 O-glycoforms and find that two major glycoforms, HexNAc-Gal-Neu5Ac and HexNAc-Gal-2Neu5Ac, represent approximately 85 % of the total. Additional minor O-glycoforms represent multiplication of the same structures attached to additional serine/threonine residues of the same tryptic peptide [26]. Contrary to the increased occupancy of the additional O-glycosites of HPX observed in congenital disorders of glycosylation [26], we do not observe changes in occupancy of additional sites in liver disease but observe increased sialylation of the O-glycan attached to the N-terminal threonine (T1) of HPX. It is important to note that the increase in sialylation is specific to the O-glycans of HPX. We have examined N-glycopeptides in the tryptic digests of HPX simultaneously and do not observe increase in their sialylation except for an approximately 30 % increase in case of one of the N-glycopeptides (Additional file 1). This is marginal compared to the several-fold increase in the di-sialylated O-glycoform of HPX observed in the same samples (Fig. 2).

We have therefore developed targeted LC–MS3 and LC–MS/MS-MRM assays for quantification of the di-sialylated and mono-sialylated glycoforms [12] and we use the assays, adapted to the analysis on the 6500 QTRAP mass spectrometer, to determine S-HPX (ratio of the two glycoforms) in a pilot study of HALT-C participants. Analysis of the results using the two targeted methods shows that quantification of S-HPX by LC–MS/MS-MRM directly in serum has lower variability (RSD = 6 % in repeated analysis of a QC sample) compared to the LC–MS3 assays (RSD = 18 %) and has sufficient sensitivity on the 6500 QTRAP instrument to achieve quantification in all samples examined. We have therefore used the LC–MS/MS-MRM assay for the final quantification of S-HPX.

Our results show that S-HPX increases progressively in fibrotic and cirrhotic participants compared to disease-free controls (Fig. 3). The LC–MS/MS-MRM assay has adequate analytical performance for direct quantification of S-HPX in serum samples which could facilitate its development into a reliable clinical test. The combined novelty of monitoring specific O-glycoforms of HPX by the newly optimized LC–MS/MS-MRM workflow makes our quantification of S-HPX in liver disease quite unique and efficient.

At this point, we do not know the mechanism responsible for the increase of S-HPX in liver disease; further studies will be needed to explore the formation and degradation of these sialylated O-glycoforms of HPX. While IFN treatment seems to lower somewhat S-HPX in the HALT-C participants, this trend is not significant (Additional file 1) and does not decrease the ability of S-HPX to distinguish fibrotic and cirrhotic disease. IFN treatment, especially in patients achieving a sustained viral response, reduces risk of HCC [40] but maintenance treatment with pegylated IFN was not found effective in the HALT-C trial [41, 42]. Since the IFN treatment is not expected to be clinically relevant, we did not further study its effect. To generate leads for further exploration, we correlated the appearance of S-HPX with available demographic and clinical factors. These associations show that S-HPX correlates with AFP, ALT, AST, INR and bilirubin and correlates negatively with albumin and platelet count (Table 3). AFP and platelet count are established measures of liver disease and HCC. Previous analyses of the HALT-C trial [43] and other populations [44] showed that serum AFP is elevated in patients with advanced chronic hepatitis C, with or without HCC, especially among female and African American patients [45]. Platelet count [46] and the APRI [47] or FIB-4 [48] indexes, which combine platelet count with AST and ALT, have been associated with the degree of fibrosis and disease severity in the HALT-C study. These factors appear to be regulated in a similar way as S-HPX; however, S-HPX performs comparably well or better than any of these measures in our study. Further studies in larger population samples are required to establish definitive cut-offs of S-HPX and to validate diagnostic models in combination with other variables.

## Conclusion

In conclusion, we have detected, for the first time, elevation of di-sialylated O-glycoform of HPX in liver disease and established LC–MS/MS-MRM assay for its quantification in serum. We propose that this measure could serve as a simple indicator of liver fibrosis alone or in combination with other factors currently used to classify liver disease. Analysis of the sialylated glycoform at a specific site of HPX attachment directly in unfractionated serum facilitates clinical applicability of the robust MRM quantification method. The pathophysiological mechanisms leading to increased S-HPX and definitive assessment of its behavior in liver disease of HCV and other etiologies will require further studies.

## Additional file

10.1186/s12014-016-9125-x. Supplemental methods, figures and tables. **SM 1.1.** Mapping of the O-glycosylation sites. **SM 1.2.** Beta elimination and mass spectrometric analysis of HPX O-glycans. **Table S1.** Basic characteristics of disease-free controls and HALT-C participants. **Table S2.** The impact of IFN treatment on S-HPX. Groups of fibrotic and cirrhotic participants in the HALT-C trial were separated into the IFN treated and control arms. **Table S3.** Model estimates for logistic regression model in discovery set. **Table S4.** S-HPX measurement in the discovery and validation sets of participants. **Figure S1.** Precursor mass spectra confirmation of complete desialylation of HPX using 2M Acetic acid. **Figure S2.** ETD spectra of sialidase-treated O-glycopeptides corresponding to HILIC fractions of mono- (*top*), bis- (*middle*), and triply-glycosylated (*bottom*) O-glycopeptide of HPX. **Figure S3.** Direct quantification of S-HPX at progressing stages of liver disease divided by gender (*left*) and race (*right*; *CA* Caucasian, *AA* African-American). **Figure S4.** Significant associations of S-HPX and other clinical variables. **Figure S5.** Quantification of detected N-glycopeptides at three different N-glycosylation sequons (N64, N187 and N453) of HPX.

## Abbreviations

AA: African American
AFP: alpha-fetoprotein
ASN: asparagine
ALT: alanine transaminase
AST: aspartate transaminase
CA: Caucasian American
CE: collision energy
CID: collision induced dissociation
CDG: congenital disorders of glycosylation
Disialylated-HPX: (NeuAc)_2_(Hex)_1_(HexNAc)_1_-TPLPPTSAHGNVAEGETKPDPVTER
DP: declustering potential
ER: endoplasmic reticulum
GalNAc: N-acetylgalactosamine
HALT-C: hepatitis C antiviral long-term treatment against cirrhosis trial
HCC: hepatocellular carcinoma
HCV: hepatitis C virus
HEX: hexose
HexNAc: N-acetyl hexosamine
HILIC: hydrophilic interaction chromatography
HP: haptoglobin
HPX: hemopexin
IAA: iodoacetamide
IFN: interferon
INR: international normalized ratio for prothrombin time
LC-ETD/MS: liquid chromatography electron transfer dissociation mass spectrometry
LC/MS3: liquid chromatography with triple-stage mass spectrometric detection
LIT: linear ion trap
MELD: model for end-stage liver disease
Monosialylated-HPX: (NeuAc)_1_(Hex)_1_(HexNAc)_1_-TPLPPTSAHGNVAEGETKPDPVTER
MRM: multiple reaction monitoring
MS1: precursor ion mass spectrometry
MS2: CID product ion mass spectrometry
NAFLD: nonalcoholic fatty liver disease
NASH: nonalcoholic steatohepatitis
NeuAc: N-acetylneuraminic acid
NIDDK: National Institute of Diabetes and Digestive and Kidney Diseases
QqQ: triple quadrupole mass spectrometer
Pro: proline
S-HPX: the ratio of disialylated-HPX/monosialylated-HPX
SRM: selected reaction monitoring
UPLC: ultra performance liquid chromatography
XIC: extracted ion chromatogram
