# Supplementary material for: Increased sialylation of site specific O-glycoforms of hemopexin in liver disease
Source: Clin Proteomics. 2016 Sep 21;13:24. doi: 10.1186/s12014-016-9125-x (PMC5034550; doi:10.1186/s12014-016-9125-x)

## Supplemental Methods, Figures and Tables

**SM 1.1 Mapping of the O-Glycosylation Sites:** Tryptic digest of HPX was labeled by mTRAQ (AB Sciex, Framingham, MA) reagent Δ4 tag according to manufacturer's protocol and analyzed by LC-MS/MS-ETD on an Orbitrap XL mass spectrometer (Thermo-Fisher, San Jose, CA). mTRAQ Δ4-labeled tryptic peptides, de-sialylated tryptic peptides and de-sialylated/de-galactosylated tryptic peptides were loaded onto a 75 μm x 8.5 cm C18 reverse phase column (YM C GEL ODS-AQ120AS-5) using nitrogen pressure. Peptides were separated over a 100 min linear gradient from 5-100% Solvent B with a flow rate of 250 nL/min and analyzed on a linear ion trap mass spectrometer (LTQ Orbitrap XL, Thermo Scientific) using a method composed of full scan, 300-2,000 m/z, and targeted analysis of mTRAQ Δ4-labeled O-glycopeptides, 200-2,000 m/z, in a 3Da isolation window and 90 ms reaction time. The ion source capillary temperature was set to 200°C with a capillary voltage of 46 V and a tube lens voltage of 120 V.

**SM 1.2 Beta elimination and mass spectrometric analysis of HPX O-glycans:** Isolated HPX (20 μg) was cleaved by reductive beta-elimination. Samples were treated with 1 M $NaBH_4$ in 50 mM NaOH for 18 h at 45°C, neutralized by addition of 10% acetic acid, desalted using an AG50-X8 (Bio Rad) cartridge, and eluted with 5% acetic acid. Borate was removed by re-suspending the dried sample in 9:1 methanol/acetic acid followed by four cycles of drying under a stream of nitrogen at 37°C. Detached glycans were then permethylated, permethylated glycans were dissolved in 1 mM NaOH/50% MeOH and directly infused via nano-spray emitter onto a tandem mass spectrometer (LTQ Orbitrap XL, Thermo Scientific) at a flow rate of 0.4 μL/min for manual MS/MS analysis. Intact permethylated O-glycans were analyzed via FTMS in the range of *m/z* 300-2,000, and MS/MS spectra were acquired from *m/z* 50-2,000.

**Table S1:** Basic characteristics of disease-free controls and HALT-C participants [a]

| Discovery | Healthy | Fibrosis | Cirrhosis | p-value [b] |
|---|---|---|---|---|
| N | 23 | 22 | 24 | n/a |
| male [%] | 65 | 50 | 71 | 0.326 |
| race (CA/AA) | 8/9 [c] | 18/4 | 16/8 | 0.019 |
| age | 48.4 ± 9.2 | 51.0 ± 7.5 | 50.3 ± 7.5 | 0.578 |
| IFN treatment (yes/no) | n/a | 13/9 | 12/12 | 0.536 |
| ALT [U/L] | n/a | 102.7± 66.8 | 122 ± 71.8 | 0.231 |
| AST [U/L] | n/a | 72.9 ± 35.5 | 109.6 ± 77.3 | 0.126 |
| platelet ($10^9$ / L) | n/a | 196.4 ± 55.4 | 143.3 ± 66.0 | <0.001 |
| albumin [g/dL] | n/a | 4.0 ± 0.4 | 3.6 ± 0.5 | 0.016 |
| AFP [ng/mL] | n/a | 9.7 ± 12.0 | 20.1 ± 16.8 | 0.002 |
| alkaline phosphatase [U/L] | n/a | 81 ± 33 | 131 ± 92 | 0.553 |
| INR | n/a | 1.0 ± 0.1 | 1.2 ± 0.2 | <0.001 |
| bilirubin [mg/dL] | n/a | 0.8 ± 0.6 | 1.3 ± 0.8 | 0.057 |
| creatinine [mg/dL] | n/a | 0.9 ± 0.3 | 0.9 ± 0.3 | 0.301 |
| MELD | n/a | 7.6 ± 2.2 | 10.0 ± 2.9 | 0.009 |
| APRI | n/a | 0.9 ± 0.5 | 2.6 ± 2.8 | 0.004 |
| FIB-4 | n/a | 1.7 ± 0 .6 | 3.66 ± 3.12 | 0.006 |

| Validation | Healthy | Fibrosis | Cirrhosis | p-value [b] |
|---|---|---|---|---|
| N | 15 | 15 | 15 | n/a |
| male [%] | 73 | 67 | 73 | 0.993 |
| race (CA/AA) | 12/3 | 12/3 | 12/3 | n/a |
| age | 50.6 ± 4.8 | 48.3 ± 6.8 | 51.5 ± 4.7 | 0.281 |
| IFN treatment (yes/no) | na | 0/15 | 0/15 | n/a |
| ALT [U/L] | na | 81.4 ± 32.5 | 133 ± 90.7 | 0.169 |
| AST [U/L] | na | 64.4 ± 25.3 | 110 ± 58.5 | 0.040 |
| platelet ($10^9$ / L) | na | 179 ± 57.3 | 153 ± 36.2 | 0.266 |
| albumin [g/dL] | na | 4.0 ± 0.4 | 3.9 ± 0.4 | 0.616 |
| AFP [ng/mL] | na | 13.3 ± 17.1 | 26.3 ± 33.5 | 0.396 |
| alkaline phosphatase [U/L] | na | 94 ± 26 | 104 ± 60 | 0.967 |
| INR | na | 1.0 ± 0.1 | 1.0 ± 0.1 | 0.129 |
| bilirubin [mg/dL] | na | 0.7 ± 0.3 | 0.8 ± 0.4 | 0.595 |
| creatinine [mg/dL] | na | 0.8 ± 0.2 | 0.9 ± 0.2 | 0.416 |
| MELD | na | 6.3 ± 0.6 | 7.3 ± 1.5 | 0.012 |
| APRI | na | 0.1 ± 0.6 | 1.8 ± 1.2 | 0.050 |
| FIB-4 | na | 2.2 ± 1.3 | 3.4 ± 1.4 | 0.050 |

[a] Values are expressed as mean ± standard deviation
[b] *p*-value was calculated using Mann-Whitney U test or Kruskal-Wallis test for quantitative variables and chi-square test for categorical variables.
[c] six healthy controls belong to the Hispanic ethnic group

**Table S2:** The impact of IFN treatment on S-HPX. Groups of fibrotic and cirrhotic participants in the HALT-C trial were separated into the IFN treated and control arms.

| **Group (N)** | **IFN** | **Mean ± Std** | **Median (Min, Max)** | ***p*-value** |
|---|---|---|---|---|
| FIB (13) | yes | 20.9 ± 8.1 | 20.6 (11.0, 41.3) | 0.161 |
| FIB (9) | no | 30.7 ± 19.1 | 25.0 (13.8, 76.0) | |
| CIR (12) | yes | 55.4 ± 30.0 | 48.0 (19.5, 106.5) | 0.157 |
| CIR (12) | no | 81.7 ± 42.7 | 78.8 (22.1, 156.1) | |

**Table S3:** Model estimates for logistic regression model in discovery set.

| | $\hat{\alpha}$ | | | $\hat{\beta}$ | | |
|---|---|---|---|---|---|---|
| | **Estimate** | **St. Error** | ***p*-value** | **Estimate** | **St. Error** | ***p*-value** |
| **AFP** | 0.82 | 0.521 | 0.118 | -0.054 | 0.033 | 0.099 |
| **Platelet** | -2.62 | 1.091 | 0.017 | 0.016 | 0.006 | 0.013 |
| **APRI** | 2.01 | 0.805 | 0.013 | -1.500 | 0.646 | 0.020 |
| **FIB-4** | 2.27 | 0.869 | 0.009 | -0.805 | 0.318 | 0.011 |
| **S-HPX** | 2.84 | 0.884 | 0.001 | -0.076 | 0.025 | 0.002 |

**Table S4:** S-HPX measurement in the discovery and validation sets of participants

| Discovery | N | Mean ± SD | Median (Range) | p-value |
|---|---|---|---|---|
| CTRL | 23 | 14.7 ± 6.67 | 13.3 (4.81,32.0) | <0.001 |
| FIB | 22 | 24.9 ± 14.2 | 21.0 (11.0,76.0) | |
| CIR | 24 | 68.6 ± 38.5 | 65.3 (19.5,156.1) | |

| Validation | N | Mean ± SD | Median (Range) | p-value |
|---|---|---|---|---|
| CTRL | 15 | 16.2 ± 7.36 | 15.0 (7.91,32.8) | <0.001 |
| FIB | 15 | 25.8 ± 11.1 | 23.5 (15.2,60.0) | |
| CIR | 15 | 54.9 ± 38.9 | 46.4 (13.1,177.6) | |

**Figure S1:** Precursor mass spectra confirmation of complete desialylation of HPX using 2M Acetic acid

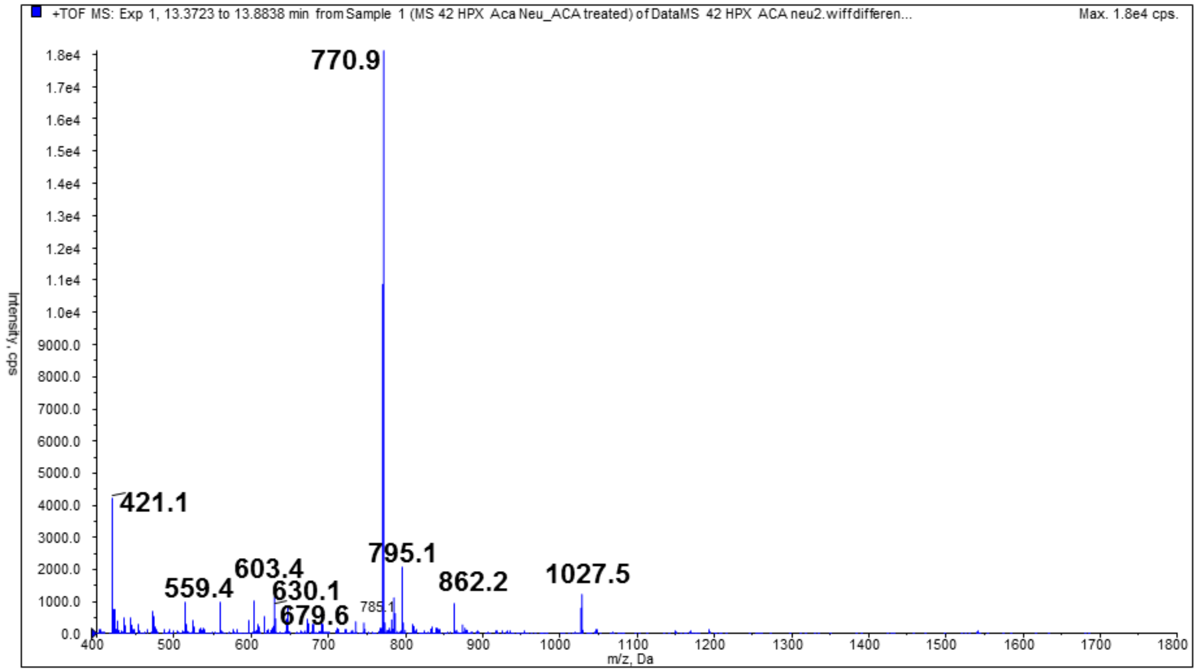

**Figure S2:** ETD spectra of sialidase-treated O-glycopeptides corresponding to HILIC fractions of mono- (top), bis- (middle), and triply- glycosylated (bottom) O-glycopeptide of HPX. The labeled fragments (C2, C3, C6, C7 and C8) define unequivocally the sites occupied by O-glycans.

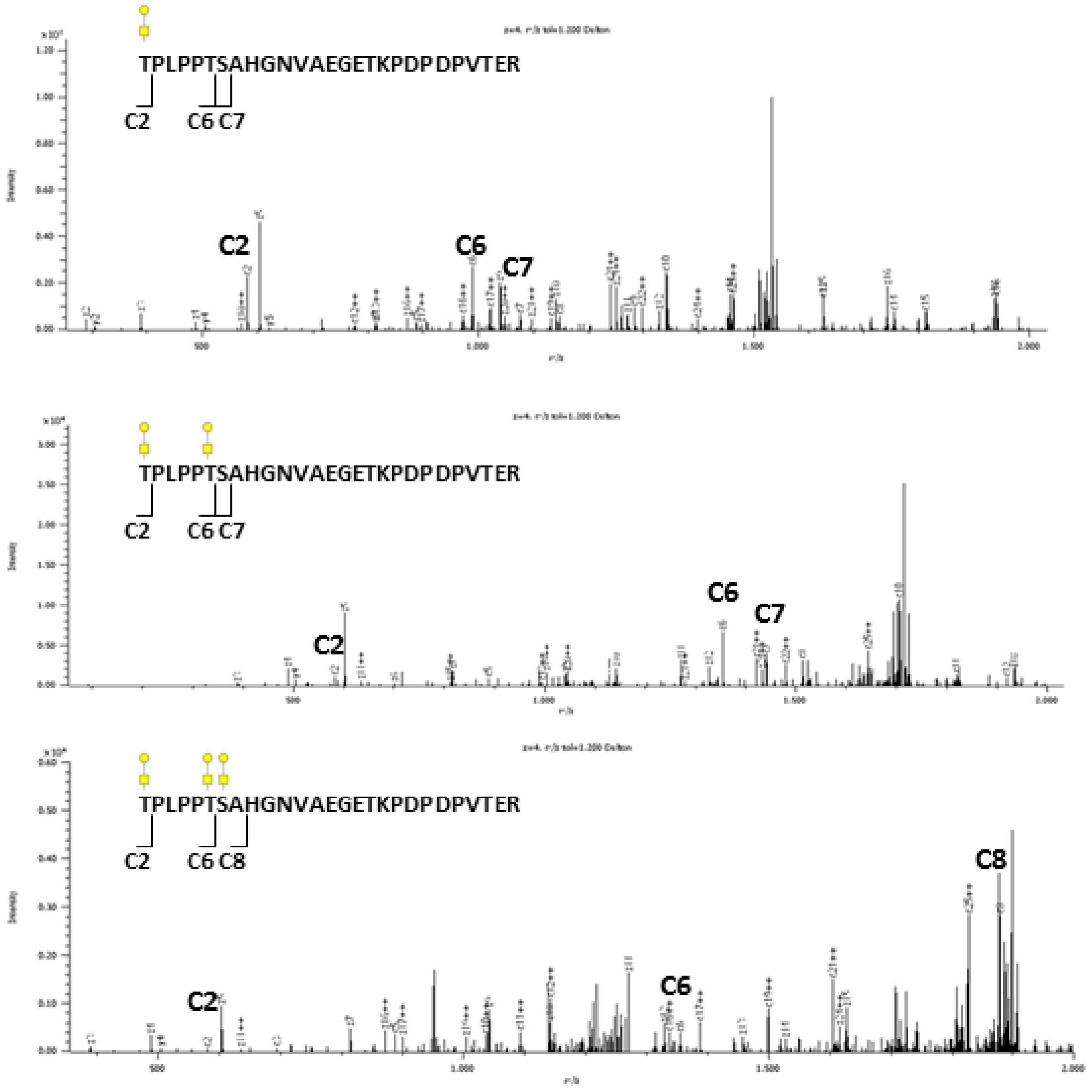

**Figure S3:** Direct quantification of S-HPX at progressing stages of liver disease divided by gender (left) and race (right; CA – Caucasian, AA- African-American)

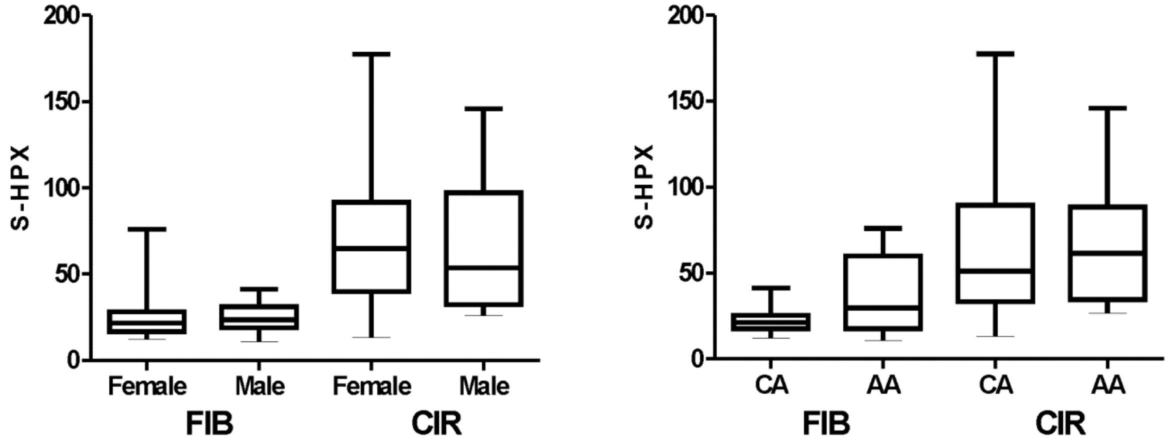

**Figure S4:** Significant associations of S-HPX and other clinical variables (bilirubin, platelets, AFP, albumin, ALT, AST, APRI, FIB-4) in the groups of fibrotic (open symbols, n=22) and cirrhotic (filled symbols, n=24) participants.

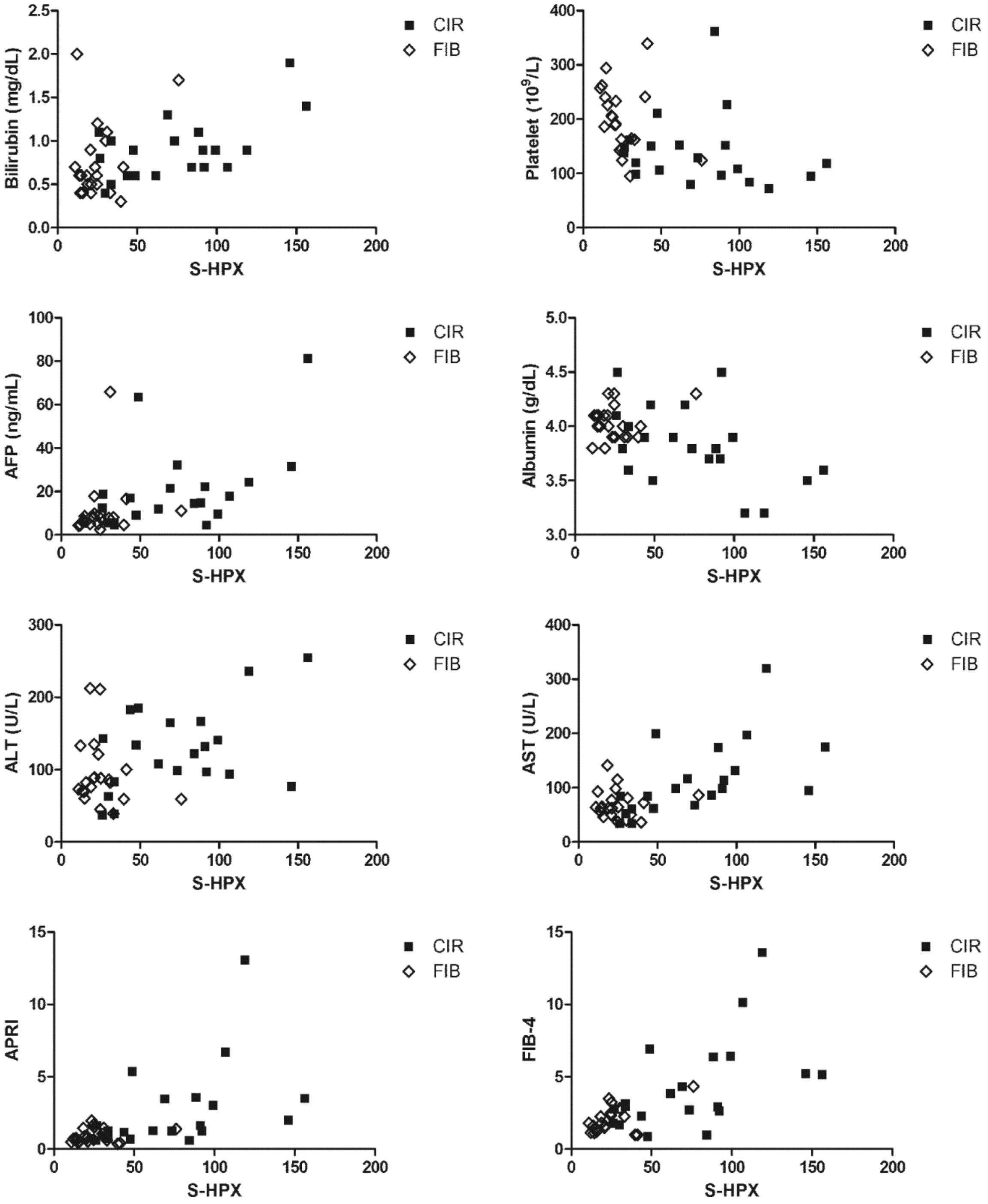

**Figure S5:** Quantification of detected N-glycopeptides at three different N-glycosylation sequons (N64, N187 and N453) of HPX isolated from pooled plasma of healthy volunteers (white bars), cirrhotic (grey bars), and HCC patients (black bars). Results represent XIC of precursor ions normalized to an internal peptide of HPX and error bars represent parallel analysis of two different plasma pools (samples of 5 participants in each pool) as described in methods. AxGySz denotes the following: number of antennas (A), galactoses (G), and sialic acids (S) associated with each sequon.

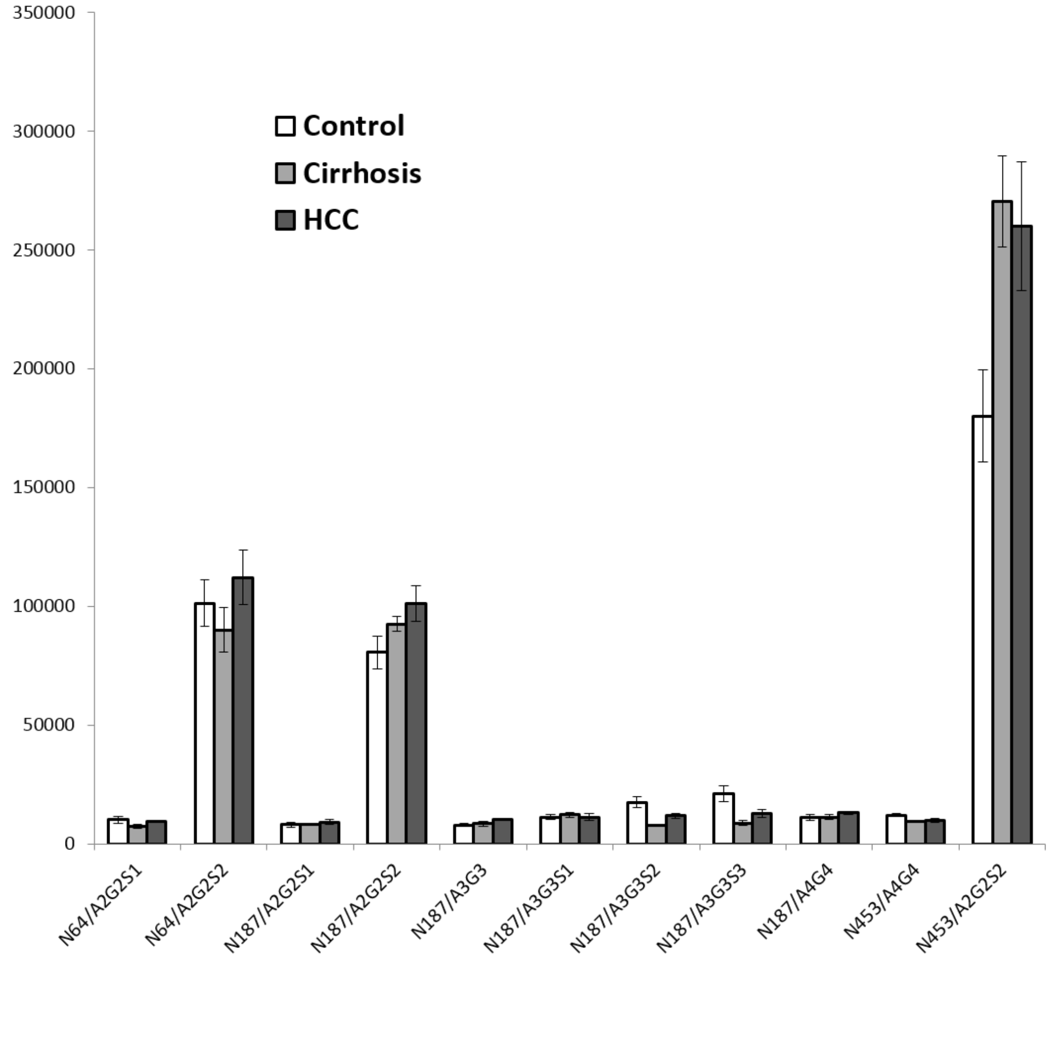

Supplement: Supplementary file 1 — 10.1186/s12014-016-9125-x. Supplemental methods, figures and tables. SM 1.1. Mapping of the O-glycosylation sites. SM 1.2. Beta elimination and mass spectrometric analysis of HPX O-glycans. Table S1. Basic characteristics of disease-free controls and HALT-C participants. Table S2. The impact of IFN treatment on S-HPX. Groups of fibrotic and cirrhotic participants in the HALT-C trial were separated into the IFN treated and control arms. Table S3. Model estimates for logistic regression model in discovery set. Table S4. S-HPX measurement in the discovery and validation sets of participants. Figure S1. Precursor mass spectra confirmation of complete desialylation of HPX using 2M Acetic acid. Figure S2. ETD spectra of sialidase-treated O-glycopeptides corresponding to HILIC fractions of mono- (top), bis- (middle), and triply-glycosylated (bottom) O-glycopeptide of HPX. Figure S3. Direct quantification of S-HPX at progressing stages of liver disease divided by gender (left) and race (right; CA Caucasian, AA African-American). Figure S4. Significant associations of S-HPX and other clinical variables. Figure S5. Quantification of detected N-glycopeptides at three different N-glycosylation sequons (N64, N187 and N453) of HPX. [file 12014_2016_9125_MOESM1_ESM.pdf]
